# Imaging Inflammation and Infection in the Gastrointestinal Tract

**DOI:** 10.3390/ijms21010243

**Published:** 2019-12-30

**Authors:** Alex N. Frickenstein, Meredith A. Jones, Bahareh Behkam, Lacey R. McNally

**Affiliations:** 1 Stephenson School of Biomedical Engineering, University of Oklahoma, Norman, OK 73019, USA; africk256@ou.edu (A.N.F.); meredith.jones@ou.edu (M.A.J.); 2 Stephenson Cancer Center, University of Oklahoma, Oklahoma City, OK 73104, USA; 3 Department of Mechanical Engineering, Virginia Tech University, Blacksburg, VA 24061, USA; behkam@vt.edu; 4 Department of Surgery, University of Oklahoma, Oklahoma City, OK 73104, USA

**Keywords:** gastrointestinal tract, inflammation, infection, molecular imaging

## Abstract

A variety of seemingly non-specific symptoms manifest within the gastrointestinal (GI) tract, particularly in the colon, in response to inflammation, infection, or a combination thereof. Differentiation between symptom sources can often be achieved using various radiologic studies. Although it is not possible to provide a comprehensive survey of imaging gastrointestinal GI tract infections in a single article, the purpose of this review is to survey several topics on imaging of GI tract inflammation and infections. The review discusses such modalities as computed tomography, positron emission tomography, ultrasound, endoscopy, and magnetic resonance imaging while looking at up-an-coming technologies that could improve diagnoses and patient comfort. The discussion is accomplished through examining a combination of organ-based and organism-based approaches, with accompanying selected case examples. Specific focus is placed on the bacterial infections caused by *Shigella spp.*, *Escherichia coli,* *Clostridium difficile*, *Salmonella*, and inflammatory conditions of diverticulitis and irritable bowel disease. These infectious and inflammatory diseases and their detection via molecular imaging will be compared including the appropriate differential diagnostic considerations.

## 1. Introduction

Gastrointestinal (GI) tract disorders are comprised of a wide variety of infectious, inflammatory, and malignant diseases, many of which manifest with similar clinical symptoms including abdominal pain and diarrhea. Abdominal pain is the most common reason for an emergency department visit, is on the rise and comprises 8.6% of adult and 14.0% in pediatric emergency department visits [1,2,3,4]. Identification of the etiology of abdominal pain is based primarily on structural differences with little knowledge about the molecular characteristics of the affected tissues, resulting in high rates of misdiagnosis and improper treatment. Along with similar physical presentations, many GI diseases also have similar bio-clinical symptoms such as, leukocytosis and fevers which are extremely nonspecific, providing little to no insight for a proper diagnosis [5]. At present, the ability to locally identify inflammation and infection within the GI tract is confined to anatomic methods. While molecular imaging may fill this gap in the future, its ability to identify a specific genus of bacterial organism, separate Gram-positive/-negative bacteria, or recognize specific bacterial strains may be limited largely due to a lack of development or translation of bacterial strain specific contrast agents. Likewise, the use of laboratory and ex vivo methods, mostly blood or stool tests, have the capability to identify specific species with potential subspecies or strain, but also generally lack the capability to localize the site of infection.

There is currently no gold standard of care for a patient presenting with symptoms consistent with a GI illness. Cross sectional imaging techniques have started being used to aid in GI malady analysis, optimizing treatment plans through earlier and more accurate detection of disease [6]. Drastically different treatment protocols must be followed for GI infections, inflammation, and cancer, so it is imperative that disease’s causative agents are identified rapidly and accurately.

Diarrhea is another common and serious symptom of gastrointestinal disorders that causes up to 500,000 deaths per year [7] and is the second leading cause of death in children under the age of 5 [8]. Developing nations see a higher rate of deaths due to GI diseases than developed nations which may be attributed to the difference in standard of care and available treatments. Bacterial infections are frequently the causative agents of diarrhea, all which require highly specific in vitro procedures that require a lab and expensive equipment. These facilities may not be readily available in developing areas, leading to a higher misdiagnosis rate and improper treatment of diseases. Inflammation is often associated with these bacterial infections, making it even more difficult to differentiate between infections and inflammatory conditions that both cause abdominal pain and diarrhea. 

Imaging is a promising next step for the differentiation of nonspecific infectious and inflammatory symptoms in the GI tract. For this review of GI imaging, prevalent infections and inflammatory conditions of the GI tract are discussed. To provide an appropriate background on each disease, their symptoms and epidemiology are briefly detailed. The prevalence and usefulness of various imaging modalities for each disease is then considered. A summary of the specificity and selectivity results obtained from a variety of human and animal studies on is listed in Table 1. Finally, identification and differentiation of discussed maladies from GI malignant tumors is reviewed before analyzing future directions for the field.

## 2. Infection

Early or chronic infections are difficult to detect. Non-invasive diagnosis of infectious and in particular discrimination between infection and other sources would have a profound impact on disease management and patient outcomes. To this end different imaging techniques such as radiograph, ultrasonography, magnetic resonance imaging (MRI), positron emission tomography (PET), computed tomography (CT), and single photon emission computed tomography (SPECT) have been explored [22,23,24,25,26]. The morbidity and mortality rate associated with diarrhea in immunocompromised patients is much higher than that in a standard adult due to their suppressed immune systems. Ninety-five percent of AIDS patients in developing countries had acquired chronic diarrhea [27]. Along with immunocompromised patients, children are also at a higher risk for contracting GI infections due to underdeveloped immune system and frequent oral contact with foreign, unsanitary objects. The prevalence of these infectious diseases in underdeveloped nations due to poor sanitation and over crowdedness is a major public health problem [28].

MRI enables diagnosis of microbial infection and has also been used for longitudinal bacteria tracking in vivo. MRI diagnostics have potential for translation into clinical use. Application of MRI for diagnosis of bacterial and viral infectious diseases is currently limited to the detection of the resulting local inflammation, edema formation and the resulting changes in the local tissue properties (e.g., relation time, water content, and diffusivity), as well as other manifestations of the immune response, and not the direct detection of the causative infectious agent [29]. 

PET detects the dissemination of pathogens indirectly via detection of changes in cellular processes and metabolic turn over, therefore, it cannot detect between infection and inflammation [26,30]. Exogenously radiolabeled patient leukocytes, as well as ^99m^ Tc, ^67^Ga, and ^18^F-FDG tracers have been utilized for clinical PET imaging due to their absorbance by cells exhibiting high metabolic rates [31]. However, specific radiolabeled antimicrobial peptides, antibodies, and antibiotics have been proposed for specific detection of infection via SPECT. Although given the low resolution of this technique, further improvements are needed [32].

Ultrasonography (US) is used to detect pathological changes in tissue due to infection. Similar to the previous imaging modalities, US is unable to detect the causative infectious agent. For instance, ultrasonography has been used to detect salmonella enterocolitis through detection of pathological changes in bowel, ascites, and colonic wall thickening which may provide insight into the severity of infection [33].

White light imaging (WLI) and linked color imaging (LCI) endoscopy have also used for diagnosis of active infections. For example, *Helicobacter pylori* infection identification using WLI was shown to have a sensitivity and specificity of 81.7%, and 66.7%, respectively, while significantly higher sensitivity and specificity of 93.3%, and 78.3% were reported for LCI [34].

### 2.1. Shigella spp.

*Shigella spp.* is a Gram-negative bacterial pathogen, that is primarily transmitted via the oral–fecal route [28,35,36,37,38]. Out of 165 million cases of *Shigella* annually, 1.5 million cases resulted in fatalities with 98% being in underdeveloped nations [39] and approximately 500,000 cases reported in the United States alone [40]. Pathogenesis of *Shigella* causes dysentery accompanied by vomiting, dehydration, and abdominal pain. Colonic inflammation is seen in shigellosis, but this inflammation alone is not specific enough to diagnose the patient. Blood and mucous in the feces is a good indication of *Shigella*, but further testing is required to identify the correct pathogen and administer proper treatment [41]. The presence or absence of the *Shigella* pathogen is traditionally identified using a variety of techniques in stool (Table 2), a difficult, time consuming, and expensive process [28,35,41]. Alternative in vitro techniques have been utilized to identify *Shigella* in stool cultures.

The *Shigella* species has 4 subtypes, *S. dysenteriae*, *S. flexneri*, *S.boydii*, and *S. sonnei*. All four types contain a virulent plasmid that is around 120–140 mDal in size. *Shigella* specific DNA probes have been derived and used successfully to identify the presence of the pathogen [47]. The DNA is either extracted from a stool sample and amplified by polymerase chain reaction to accumulate quantities that can be detected by DNA probes or stool blots can be treated with the DNA probes [28,41]. An alternative to DNA probing is the use of an enzymatically-linked immunosorbent assay (ELISA) to identify the pathogen in vitro. The *Shigella* plasmid encodes for virulent antigens, including invasion plasmid antigen (Ipa) proteins and a covalently linked extracellular lipopolysaccharide O-antigen, both of which can be identified by an ELISA with an appropriate antiserum [35,42]. The combination of DNA probes and ELISA can provide an accurate diagnosis of *Shigella*, but are not readily available from a clinical standpoint [28]. 

### 2.2. Escherichia coli

While Escherichia coli has many beneficial functions in the GI tract, there are many different diarrheagenic forms of the bacteria that are very dangerous in. Enteroinvasive Escherichia coli (EIEC) is the pathogenic form that is indistinguishable from *Shigella* on the species level and induces the same clinical symptoms as *Shigella*. Like *Shigella*, EIEC is also transmitted via the fecal-oral route and is responsible for up to 40% of diarrhea in children under the age of 5 [30]. The taxonomical relatedness of the two pathogens makes it very difficult to differentiate between them, thus identify the correct etiological agent without a gold-standard test (Table 2) [30,41].

Both EIEC and *Shigella spp*. contain a large virulence plasmid that codes for similar invasion plasmid antigen proteins and O-antigens [48]. Serotyping of the O-antigens had been used as the gold standard to differentiate between *E. coli* serotypes, but this procedure is often erroneous and require extensive lab technique. DNA probing and multiplex PCR assays of stool samples targeting the O and H antigens along with the virulent plasmids are more specific ways that have been successfully utilized to positively identify the pathogen [49]. A recent study done by Pautureau et al. explored nuclear magnetic resonance in order to differentiate between *Shigella* and EIEC. An analysis of untargeted proton NMR metabolomics was able to successfully differentiate between the two bacteria based on the metabolic footprint produced [44]. NMR’s characterization of the metabolites used by the *Shigella* bacteria and EIEC pathogen can also provide insight into better ways to identify and treat the infection. 

### 2.3. Clostridium Difficile

Usually spread through the fecal-oral route, *Clostridium difficile* causes such nonspecific symptoms as diarrhea, colitis, abdominal pain, and possibly fever or shock. The diarrhea from the disease is often associated with antibiotic therapy in the weeks preceding infection [50]. Annually, it is estimated that there are 453,000 infections and 29,000 deaths associated with *C. difficile* [51,52,53]. From these figures alone, the morbidity of *C. difficile* infection (CDI) is apparent, made even more so by a study that revealed that 9% of patients admitted to hospitals for CDI will die [54]. The startling mortality of CDI is due in part to greater prevalence of fulminant colitis [51,53,55]. Rapid identification and treatment, usually by antibodies, is necessary to increase likelihood of patient survival. At present, stool collection and testing is a common method for CDI diagnoses. Specifically, the gold standard is to test for the presence of toxins A and B in patient feces in vitro (Table 2) [51,52,56]. Testing for the presence of *C. diff* alone is not sufficient for diagnoses as a) *C. diff* is innately present in the gut microbiomes of 4% of healthy adults and b) some strains of *C. diff* do not produce toxins [52]. Toxin presence can be determined in vitro through multiple protocols, though the current best practice is to use toxigenic culture testing [46,52]. While highly accurate, the test can take between 2 and 5 days to complete as *C. diff* needs to be isolated and grown from patient stool samples and requires facilities with the appropriate instrumentation [46,50]. 

**Figure 1:**
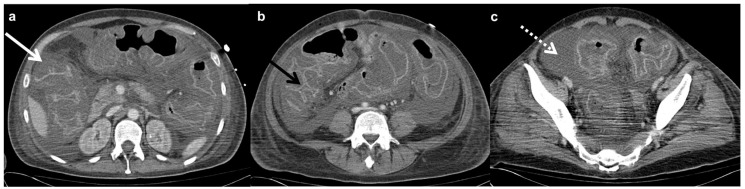
CT imaging used to identify *C. diff.* with an axial CT scan demonstrates low-attenuation wall thickening involving the entire length of the colon with intense mucosal enhancement (white arrow, (**a**)); the enhancing mucosa is stretched over the edematous haustral folds, resembling an accordion even in the absence of intra-luminal contrast (black arrow, (**b**)). A large amount of ascites is also present (dashed arrow, (**c**)) [59].

Faster methods of identification include enzyme immunoassays (EIAs) and nucleic acid amplification (NAAT). These methods bring their own complications. EIAs sensitivity values have a wide range, and both EIAs and NAAT possess difficulty in differentiating between toxic and nontoxic *C. diff* [51,52,57,58]. Fine-tuning of these methods may yield greater accuracy in CDI diagnoses. Radiologic imaging, primarily by computed tomography (CT) with oral contrast (e.g., barium sulfate) (Figure 1 [59]), may serve as an additional diagnostic tool. CT images of confirmed CDI patients show signs of bowel wall thickening, pericolonic stranding, and low-attenuation mural thickening, among others [52,59,60,61]. Ultrasonography may serve as another imaging modality to aid in confirming CDI diagnoses, according to Wiener-Well et al. [62]. Further testing of these imaging modalities as diagnostic tools for CDI is recommended before clinical action is taken.

### 2.4. Salmonella

As with most diseases of the GI tract, infection by nontyphoid *Salmonella enterica* (NTS), referred to as salmonellosis, presents with enterocolitis and symptoms such as abdominal pain, diarrhea, and fever [62,63,64]. Most often, this bacterial infection is acquired through consuming infected foods, primarily consisting of meats, eggs, and dairy products. Globally, NTS is estimated to result in 90 million illnesses and 155,000 deaths per year [64]. In the United states, 40,000 infections and 450 deaths are reported annually as a result of NTS infection [45,64]. Children and immunocompromised youths have a higher risk of infection, with the latter being more susceptible for more severe disease manifestations or death [64]. Treatment of *Salmonella* infection often includes lessening of symptoms and administration of antibiotics [65]. To provide accurate diagnosis of salmonellosis, the current practice is to collect stool samples from patients and culture them in *Salmonella* selective media [45,64,65]. In this method, enrichment broths suited for enhanced isolation of *Salmonella* may be used to increase diagnostic accuracy [65,66]. After isolation, cell samples can be serotyped, using the Widal test, to better administer treatment and understand infection source [67,68]. Use of polyvalent and antisera, specific for the O and H antigens of individual *Salmonella* serotypes, on isolated bacteria provide necessary biochemical verification of exact pathogen identity [65].

Other methods of salmonellosis identification are possible. For example, a study by Hennedige et al. reviewed computed tomography (CT) images for confirmation of salmonellosis patients to examine radiologic manifestations of infection [69]. Of the manifestations observed, they concluded that thickened terminal ileum or thickened proximal colon with mesenteric lymphadenopathy could be specific radiologic indicators of *Salmonella* infection [69]. Further testing of these finding’s accuracy is recommended before clinical diagnoses on shigellosis by CT are performed. Due to a lack of standardization, use of ELISA testing for *Salmonella* is currently nonspecific and requires further tuning before effective use as a diagnostic tool [67,70].

**Figure 2:**
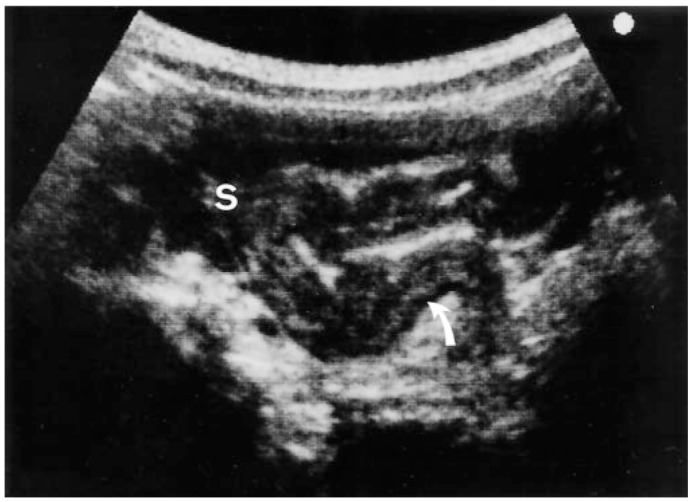
Evaluation of lower abdomen of pediatric patient with *Salmonella enterocolitis* using transverse ultrasound. There is thickening of the sigmoid colon which results predominantly from submucosal edema (arrow) observed as the thick hyperechoiclayer. S = sigmoid colon [72].

In summary, the bacterial agents responsible for GI illness along with corresponding metabolites have been successfully identified using in vitro biochemical techniques, (Table 2.) providing more information about the pathogens on a molecular level. Future implications of the in vitro work include the use of molecular imaging to identify the pathogen in vivo. Fluorescently labeled antibody/peptide (as contrast agent) for molecular imaging endoscopy for rapid detection and diagnosis of bacterial infection [71]. Ultrasounic assessment of Salmonella spp. has been successful in children with the ability to identify pathological changes in the bowel and intra-abdominal spaces with thickening of the colonic wall (Figure 2) [72]. Elimination of the need for stool cultures and biochemical laboratory procedures would drastically decrease the time take to reach a diagnosis. Molecular imaging in the GI tract is still relatively new, but has been successful in identifying malignancies and is progressing in diagnosing inflammatory and infectious diseases through the development and testing of endoscopic molecular probes [44,71,72,73].

## 3. Inflammation

### 3.1. Diverticulitis

Diverticulitis is characterized by the presence of inflamed diverticula in the GI tract, most commonly the large intestine. Sixty-five percent of the elderly population and 5% of individuals 40 years old or younger will have diverticula, 25% of those individuals will have diverticulitis while the remaining are asymptomatic [74,75]. Symptomatic diverticulitis is frequently coupled with complications including anal bleeding, fistulas, perforation, and abscess formation [74,76,77,78]. The clinical misdiagnosis rate of diverticulitis is upwards of 50%, indicating the need for better diagnostic techniques [79]. Recently there has been a spark in the investigation of potential biomarkers as safe and effective ways to diagnosis and assist physicians in treating diverticulitis [74]. C-reactive protein levels >50 mg/L, in addition to left lower quadrant abdominal tenderness and absences of vomiting is indicative of diverticulitis [79,80]. Fecal calprotectin is derived from neutrophils, so its presence in the stool is a good indication of inflammation in the GI tract. A study done by Tursi et al. showed increased levels of fecal calprotectin concentrations in diverticulitis patients when compared to IBS and control patients [81]. This provides an appealing clinical tool for differentiation between IBS and diverticulitis, although it must be noted that IBS and diverticulitis can exist simultaneously. Increased levels of these biomarkers in the blood and stool strongly support a clinical diagnosis of diverticulitis but are not specific enough to fully replace initial imaging techniques. 

#### 3.1.1. X-ray

Contrast enemas have been used in an attempt to diagnose cases of diverticulitis [82,83]. Initially, barium had been used as the contrast agent, but the shift to water soluble contrast agents was made to minimize risk to the patient. Water soluble contrast agents eliminated the risk of barium peritonitis, decreased the wait time between injection and scan, and allowed for other imaging modalities to be utilized after the enema [76,84,85]. While water-soluble contrast enemas are extremely beneficial at showing morphological changes of the colon, they are limited in that they cannot indicate active inflammation which is a key marker of diverticulitis. Therefore, the enema is then incapable of making a complete diagnosis of diverticulitis without the assistance of another imaging modality or it may result in a misdiagnosis indicating a low sensitivity of contrast enemas (Table 1) [86].

#### 3.1.2. Computed Tomography

Computed tomography (CT) has many advantages when compared to the traditional contrast enema, which is why it is now accepted as the primary imaging modality for patients that present with abdominal pain. A CT scan will identify inflamed diverticula, bowel wall inflammation, pericolic fat stranding, and corresponding complications [9,10,11,83,87,88]. CT is capable of visualizing pericolonic and colonic complications which results in a more accurate diagnosis for the patient, along with better standard of care. Thirty percent of cases of diverticulitis are accompanied by complications including, fistulas, perforations, and abscesses. Accurate assessment of complications is crucial in the development of a treatment plan. CT is particularly beneficial in the management of abscesses since it can be used for percutaneous drainage of the abscesses. Such drainage eliminates the need for multiple surgeries, making CT more cost effective and lower risk for the patients [9,10,77,82,83,86,88,89,90,91].

Contrast agents are utilized in CT scans to achieve maximum colonic distension along with colonic opacification in order to better identify inflammatory wall thickening consistent with diverticulitis [90]. Opacification of the colon is necessary to differentiate between intra and extra luminal air and fluid cavities, indicating the presence of abscesses [11,90,92]. Oral contrast agents are beneficial to the visualization of loops within the small bowel, but lack in completely covering the colon. Oral contrast agents are limited by inconsistent opacification of the colon and a large waiting period, resulting in the infrequent use of these types of agents [11,92]. Intravenous contrast agents are advantageous due to their capability of enhancing inflammation in the abdominal and pelvic regions. Such enhancement permits a more accurate identification of other illnesses that may have similar clinical symptoms as diverticulitis [83,87]. The use of an intravenous contrast agent is associated with a higher risk to the patient, at no significant benefit in comparison to rectally administered contrast agents [11,92]. Rectally administered contrast agents are the safest and most efficient way of obtaining uniform opacification of the colon with maximum distention [90]. Gastrografin-based contrast agents are the most widely used agents when imaging the gastrointestinal tract [9,11]. The risks associated with rectal contrast agents are similar to those associated with performing an enema: there is a higher chance of exacerbating perforations or extravasation of contrast material [92]. 

#### 3.1.3. Ultrasonography

Ultrasonography (US) is an extremely low cost and low risk imaging modality that is not frequently used for the diagnosis of diverticulitis due to its reliance on a technician and inferiority to CT. While ultrasound is non-invasive, provides real-time images, and is useful in identifying inflammation, abscesses, and bowel wall thickening, it is limited in that it cannot be used on obese patients and that is can be impeded by gas bubbles [77,87]. A meta-analysis of 6 CT studies and 6 US studies showed that CT was no better or worse than ultrasound at diagnosing diverticulitis (Table 1), but CT still remains the modality of choice due to its ability to detect a multitude of complications [12,77,91].

#### 3.1.4. Magnetic Resonance Imaging

While magnetic resonance imaging (MRI) is not traditionally used when a patient presents with abdominal pain, it has proven to have a high sensitivity and specificity in the diagnosis of diverticulitis, especially with the introduction of an intravenous gadolinium-based contrast agent (Table 1) [14,15,87]. MRI is superior to CT in that it lacks associated harmful ionizing radiation, thus an appealing and a safe alternative to CT, but gadolinium-based contrast agents remain somewhat controversial with potential Gadolinium toxicity [93,94]. However, the use of MRI in diagnosing diverticulitis is limited not only by its cost and time, but also by the motion of other organs to continue breathing while the scan is taking place [14]. Due to these obstacles, MRI remains in the shadow of other imaging modalities for diagnosis of diverticulitis. 

#### 3.1.5. Endoscopy

Many features that are indicative of a CT scan are also indicators of colorectal cancer. After a positive diagnosis is made using CT, it is standard procedure for the patient to get a follow up colonoscopy around 6 weeks after being diagnosed [9,95]. This is a preventative measure to ensure that the diagnosis of diverticulitis did not miss a diagnosis of colon cancer [77,82,87,91,95,96,97]. In a study conducted by Lau et al., 34% of patients’ follow up colonoscopies came back positive for further complications or misdiagnosis of the initial diverticulitis [97]. While there is nothing to indicate that a positive diagnosis for diverticulitis is correlated with a higher risk for colorectal cancer, it has been observed that patients who present with diverticular complications have been more likely to have a positive colonoscopy for colorectal cancer sometime after the diverticulitis diagnosis [77,97].

### 3.2. Irritable Bowel Disease

Irritable bowel disease (IBD) is expressed in two major forms: Crohn’s disease (CD) and ulcerative colitis (UC). For both manifestations, IBD symptoms are similar to those of other GI disorders and include abdominal pain or discomfort, weight loss, bloody stool, diarrhea, and nausea [98]. In the intestines, the small and large bowel walls thicken and abscesses, collections of fluid surrounded in the inflamed intestinal tissue, may form. Unlike other disorders, the intensity of IBD symptoms changes with time. Periods of high intensity symptoms are defined as “flare,” while times of low intensity symptoms indicate “remission” [99].

Incidence of IBD in the United States and Europe is increasing, with current estimates of the affected population in those countries exceeding 1.6 million and 3 million, respectively [99,100,101,102]. For the different manifestations of IBD, CD’s prevalence in children and adults is 58 and 241 cases per 100,000, respectively, while UC prevalence in children and adults is 34 and 263 cases per 100,000, respectively [102,103,104]. In the United States, CD incidence is currently estimated to be 3.1–14.6 cases per 100,000 person-years and UC incidence is estimated at 2.2–14.3 cases per 100,000 person-years [102,103]. IBD occurrence is spreading across the world as well, with observed incidences in developing countries [100]. Approximately 20% of IBD patients are diagnosed during their childhood [99,104]. The effects of IBD have long-term impacts on children, namely growth failure or delays in puberty onset [63,100]. In 10–15% of IBD cases, CD and UC cannot be distinguished based on how they present in the patient, who is given an “IBD-unclassified” diagnosis. IBD patients are also at an increased risk of developing colorectal cancer, primarily due to the chronic intestinal inflammation inherent to CD and UC [105,106]. Eaden et al., in their meta-analysis, showed that patients with UC developed a cumulative risk to develop colorectal cancer of 2%, 8%, and 18% at 10, 20, and 30 years, respectively, after disease development [105,106,107]. Another study by Beaugerie and Itzkowitz demonstrated that in North America and some countries in Europe, the risk of colorectal cancer in IBD patients is up to two times higher than the risk of the general public [108].

The need for rapid and accurate imaging of IBD and its manifestations is apparent given its increasing prevalence and worldwide impact. Various imaging methods are practiced for IBD identification and monitoring [99]. with potential new and more effective modalities entering the clinical landscape as time progresses.

#### 3.2.1. Endoscopy

Endoscopy is usually among the first steps carried out in diagnosing IBD [109,110,111]. The most common endoscopic technique is colonoscopy. In this procedure, clinicians insert an endoscope, comprised of a white light and a camera, into the patient via the anus for direct visualization of the colon. Colonoscopy is a highly invasive procedure, proving undesirable for patients. Other endoscopic techniques that offer minimal invasion are practiced, such as single-balloon and double-balloon enteroscopy [111].

#### 3.2.2. Chromoendoscopy

To improve the surveillance of dysplasia, lesions, and other abnormalities in mucosal topography, chromoendoscopy (CE) may be used [112,113,114]. In this technique, dilute dye (indigo carmine or methylene blue) is sprayed, within appropriate guidelines, onto the lumen of the colon using a dye spray catheter [113]. This dye better reveals the location and pattern of lesions present in the colon, aiding clinician analysis, biopsy, or removal of present tissue. Compared to standard white light colonoscopy, CE has proved to be superior in multiple studies on per-patient and per-lesion analysis [113,114]. Given this, in order to increase accuracy of analysis and dysplasia detection, most international subspecialty societies recommend the use of CE when examining IBD patients [112]. This accuracy is particularly beneficial for early detection of colorectal cancer in patients.

#### 3.2.3. Computed Tomography

Given its ease-of-access, non-invasive nature, ability to scan intraluminal and extraluminal effects, and not requiring anesthesia due to short scan times, computed tomography (CT) is a highly favorable method for IBD imaging [115]. The rapid image acquisition is particularly beneficial for pediatric patients, who may be more susceptible to the harmful effects of anesthesia. IBD evaluation via CT typically requires application of both oral and IV contrast agents, such as the gastrogafin-based agents described above [99,116,117]. These agents distend and opacify the bowel to reveal where extraluminal fluids are collected and to best characterize the abnormalities (e.g., thickening) in the bowel wall. To detect mucosal enhancement, associated with inflammation of or lesions in the intestines, CT enterography (CTE) may be used, which requires application of neutral contrast agents (e.g., water) [99,117]. Cross-sectional images of patients reveal the type and extent of their intestinal inflammation. The location and degree of inflammation and wall thickening determines the manifestation of IBD [118]. Further, these factors are heavily considered when diagnosing patients with IBD as opposed to other diseases. Specificity and sensitivity values for CT studies of IBD are shown in Table 1.

As stated, CT images can be acquired quickly and does not require use of anesthetics. Further, they are easy to reproduce and possess high spatial resolution, presenting clearer images to clinicians. These factors alone make it a strong modality for imaging IBD patients. CTE may boast greater sensitivity and specificity over magnetic resonance enterography (MRE) [99,119,120]. CT is not without its disadvantages. The greatest apparent drawback with CT usage is the required patient exposure to radiation [99,121,122]. Given the chronic nature of IBD, patients need frequent imaging for monitoring disease progress and symptom flare. Repeated exposure to ionizing radiation is not ideal, especially given the increased risk of cancer produced in patients. This is especially undesirable for pediatric patients, of which there is a significant number. Other studies have shown that CT imaging, when compared to US and MRI, may actually possess lower specificity and sensitivity for IBD diagnoses, decreasing its overall reliability [19,123]. Another potential disadvantage is that CT cannot be performed in patients who are allergic to contrast agents used.

It should be noted that the prevalence of CT usage, and thus associated regional clinician skill, is much higher in the United States as compared to Europe, where US and MRI are favored [19]. For all these modalities, this disparity can affect the specificity and sensitivity results when looking at a global as opposed to regional scale.

#### 3.2.4. Positron Emission Tomography

**Figure 3:**
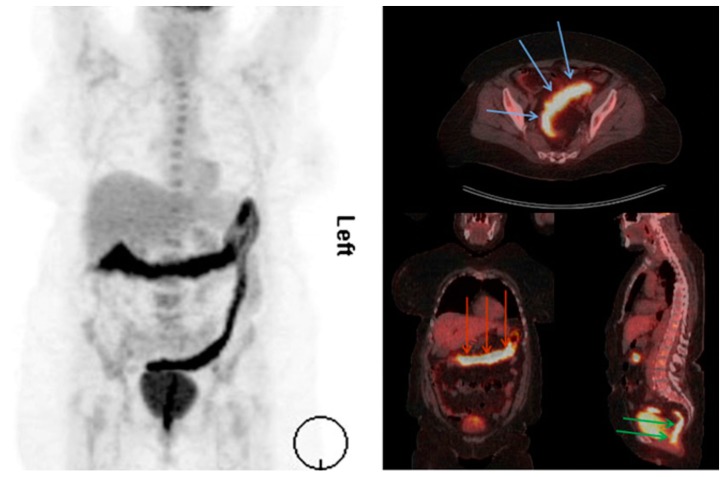
^18^F-FDG PET/CT scan of ulcerative colitis (UC) patient. Extent of disease is seen on the left and in areas indicated by arrows in the right panel [124].

For analysis of IBD, particularly in symptom flare situations or pediatric diagnostic work-ups, CT scanning is paired with positron emission tomography (PET) [21,123,124,125,126,127]. PET molecular imaging tracks accumulation of molecular radiopharmaceutical ^18^F-FDG to determine inflamed areas. ^18^F-FDG is used as it is similar to glucose and experiences significant uptake by leukocytes activated by tissue damage and inflammation. In pairing PET with CT, three-dimensional CT reconstructions follow PET molecular imaging to confirm anatomical and structural information in vivo [124]. Many studies have confirmed the usefulness of PET/CT imaging, particularly in cases where IBD affects the small bowel [123,128,129,130,131,132,133]. Further, PET/CT is reported to have better sensitivity than endoscopy, though a lower specificity than ultrasound [21,123,125]. More studies are recommended to better characterize the effectiveness of this joint modality [125,128]. An example of PET/CT imaging is shown in Figure 3.

#### 3.2.5. Immuno-PET

A relatively new modality for IBD imaging is immuno-PET. Using fragments of monoclonal antibodies (mAbs), innate immune cells, especially present during symptom flare of IBD, are targeted to track inflammation [134,135]. While testing of this modality is primarily still in the preclinical stage, murine model results are promising for translation to clinical applications [134,135]. mAb-therapeutic response can be measured with this imaging method as well [136]. This modality may also be useful in characterizing cancers of the GI tract, such as colon cancer, given the specificity of mAb technologies [136].

#### 3.2.6. Ultrasonography

Using sound waves, ultrasonography (US) provides real-time images of the body’s interior. For determining IBD presence and extent, bowel wall thickness is the primary factor considered with US modalities [99,137,138,139]. Given its non-invasive nature and ability to render images in real-time, US is heavily favored for IBD imaging and analysis. US modalities do not expose patients to any radiation, are widely available, and remain generally inexpensive [99,140]. Studied specificity and selectivity values for US usage in IBD cases can be seen in Table 1.

There are several drawbacks to US imaging for IBD. For one, US has been primarily used with CD patients, limiting its effectiveness for UC cases [137]. Further, one meta-analysis indicates US is used more in Europe than in the United States [19]. This indicates that the accuracy for US techniques, given clinician exposure and usage, may be lower in the United States in comparison to Europe. The time required and accuracy of the imaging are heavily dependent on the experience of the clinician and size of the patient. 

#### 3.2.7. Contrast-Enhanced US

Some IBD patients may still exhibit wall thickening without active inflammation [141,142,143,144]. In these cases, US could lead to inappropriate therapy for the patient as the symptom intensity misaligns with the proposed treatment. To increase the accuracy of US in these and general IBD cases, contrast enhanced ultrasonography (CEUS) may be used. CEUS uses a intravenously applied microbubble contrast agent, such as sulfur hexafluoride, to show bowel wall enhancement and mesentery [144,145]. CEUS provides real-time information on the vascularity of the scanned area. Since IBD causes vascular alterations in affected areas, this information gives clinicians an understanding on IBD activity in the patient [144,145,146,147,148]. In general, CEUS appears to provide greater clinical certainty in evaluating IBD in patients.

#### 3.2.8. Magnetic Resonance Imaging

Magnetic resonance imaging (MRI) produces three-dimensional anatomical images of patients. In imaging the GI tract, especially for small bowel analysis, MR enterography (MRE), MR enteroclysis, and MR fistulography are the commonly used procedures [99,149]. For both the small and large bowel, intra and extra mural involvement can be measured with the appropriate contrast agents such as barium suspensions [99,150]. These agents induce bowel wall distension, ensuring easier detection of bowel wall complications, and fat suppression for ease of image interpretation [99,150,151]. Fat stranding, wall thickening, and intestinal strictures are all evidence for IBD found in MRI analyses [99,152]. MRI techniques do not use ionizing radiation to acquire images, making them favorable over CT modalities, especially for young patients. Additionally, MRI produces high resolution images while permitting significantly improved soft tissue contrast as compared to CT [149].

As with previously analyzed modalities requiring contrast agents, MRI cannot be used with patients who are allergic to contrast agents. Additionally, the long scan time (compared to US, for example) is logistically inconvenient for patients, and may require sedation [99]. Further, reference information and clinician or technology-induced bias can lead to improper image assessment [19,119,143]. 

#### 3.2.9. Multispectral Optoacoustic Tomography

**Figure 4:**
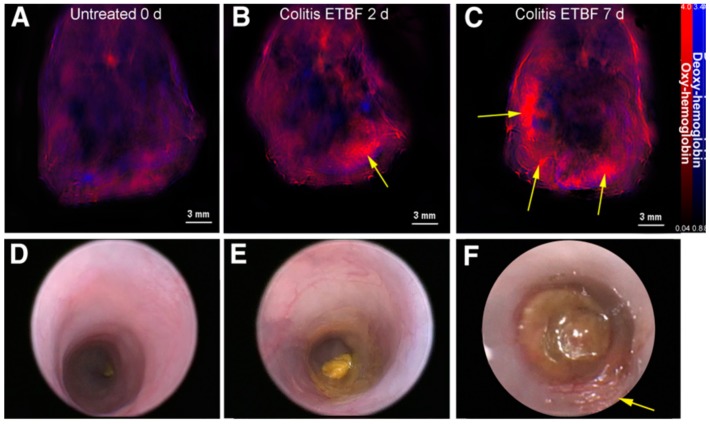
Multispectral optoacoustic tomography (MSOT) (**A**–**C**) and colonoscopic (**D**–**F**) images of murine colitis progression. Images were taken at t = 0, 2, and 7 days after bacterial inoculation. Yellow arrows indicate areas of inflammation correlating with colitis [67].

By stimulating patient tissues with lasers, ultrasound waves are generated. These waves can be read and interpreted to understand tissue characteristics inside patients. This phenomenon is the basis for multispectral optoacoustic tomography (MSOT) technologies. MSOT is purely non-invasive in nature and allows for real-time imaging of patient tissue in vivo [66,153,154]. Images gathered by MSOT show the distribution of such molecules as hemoglobin and melanin in patient tissue, due to the difference in how these molecules absorb light [155]. This distribution is indicative of disease presence and behavior in patients. A study evaluating MSOT imaging of murine colitis showed the correlation between inflammation level and concentration of oxy-hemoglobin (Figure 4) [66]. In a separate study, clinical trials on CD patients have shown similar results: Increased oxy-hemoglobin levels with active CD compared to remission CD [153,156]. MSOT technologies are still undergoing clinical trials for widespread use and are primarily seen in Europe. Preliminary results reveal significant potential for MSOT in rapidly diagnosing and monitoring IBD.

In summary, inflammatory conditions such as diverticulitis and IBD can be diagnosed and analyzed by a wide range of imaging modalities. Further work is required to verify the accuracy and refine the safety or comfortability of some of the mentioned modalities. Emerging technologies such as immuno-PET and MSOT could prove increasingly beneficial to imaging and diagnostic accuracy while increasing patient compliance. Since inflammation is also consistent with infection, it is important for patients with diverticulitis and IBD to be regularly monitored for alternative infectious or malignant diseases. 

## 4. Conclusions

Nonspecific GI symptoms can be attributed to a broad span of infectious and inflammatory conditions leading to high rates of misdiagnosis and improper patient care. Presently, anatomic imaging modalities have the capability to localize areas of inflammation with limitations in sensitivity and specificity, but lack the ability to localize specific bacterial species, albeit largely due to a lack of bacterial strain specific contrast agents. While in vitro biochemical techniques often can recognize infectious agents to correctly identify bacterial pathogens, blood or stool-based techniques often must be paired with anatomical imaging to arrive at the correct diagnosis. Future development of bacterial specific or inflammatory specific contrast agents would dramatically improve the ability to specifically and locally identify disease within the GI tract to allow for differentiation between inflammatory and autoimmune diseases in order to better diagnose and treat patients. 

## Figures and Tables

**Table 1:** Sensitivity and specificity values of different modalities in reviewed studies.

Modality	Subjects	Sensitivity	Specificity	Study
*Diverticulitis*				
CT vs. contrast enema	542 humans	98% vs. 92%	Not Reported	Ambrosetti et al. (2002) [9]
CT vs. contrast enema	56 humans	93% vs. 80%	100%	Cho et al. (1990) [10]
CT	150 humans	97%	100%	Rao et al. (1997) [11]
CT vs. ultrasound	684 humans vs. 630 humans	94% vs. 92%	99% vs. 90%	Lameris et al. (2007) [12]
Ultrasound	161 humans	98.6%	96.5%	Schwerk et al. (1993) [13]
MRI	55 humans	95%	88%	Heverhagen et al. (2008) [14]
MRI	40 humans	86%	92%	Ajaj et al. (2005) [15]
*Irritable Bowel Disease*				
MRI	50 humans	87%	88%	Ordás et al. (2013) [16]
Endoscopy	Unknown	91%	92%	Long et al. (2011) [17]
Ultrasonography	60 humans	75–96%	100%	Civitelli et al. (2014) [18]
CT	23 humans	76.9%	90%	Wold et al. (2003) [19,20]
PET/CT	65 humans	98%	68%	Lemberg et al. (2005) [21]

**Table 2:** Potential infectious agents of gastrointestinal tract.

Infectious Agent	Clinical Evaluation	Screening	Study
*Shigella*	LPS O-Antigen	ELISA/Serotyping	Lin et al. (2016) [42]; Gentle et al. (2015) [35]
*Shigella* and *Escherichia coli*	Stool blot	DNA Probing	Sethabutr et al. (1985) [43]
*Shigella* and *Escherichia coli*	Culture Media	NMR	Rautureau et al. (2019) [44]
*Salmonella*	Stool Sample	Selective Media	Gorski (2012) [45]
*Clostridium difficile*	Stool Sample	Toxigenic Culture Testing, PCR	Mirzaei (2018) [46]

## Abbreviations

GI	Gastrointestinal tract
ELISA	Enzymatically-linked immunosorbent assay
Ipa	Invasion plasmid antigen
EIEC	Enteroinvasive *Escherichia coli*
CDI	*Clostridium difficile* infection
EIA	Enzyme immunoassay
NAAT	Nucleic acid amplification
CT	Computed tomography
NTS	Nontyphoid *Salmonella enterica*
US	Ultrasonography
MRI	Magnetic resonance imaging
IBD	Irritable bowel disease
CD	Crohn’s disease
UC	Ulcerative colitis
CE	Chromoendoscopy
CTE	Computer tomography enterography
MRE	Magnetic resonance enterography
PET	Positron emission tomography
mAb	Monoclonal antibodies
CEUS	Contrast enhanced ultrasonography
MSOT	Multispectral optoacoustic tomography
